# Barriers to cancer treatment and care for people experiencing structural vulnerability: a secondary analysis of ethnographic data

**DOI:** 10.1186/s12939-023-01860-3

**Published:** 2023-03-30

**Authors:** Amber Bourgeois, Tara C. Horrill, Ashley Mollison, Leah K. Lambert, Kelli I. Stajduhar

**Affiliations:** 1 School of Nursing, University of Victoria, Institute on Aging & Lifelong Health, Stn. CSC Victoria, PO Box 1700, V8W 2Y2 Victoria, BC Canada; 2 College of Nursing, University of Manitoba, 89 Curry Place Winnipeg, R3T 2N2 Victoria, MB Canada; 3 Social Dimensions of Health, University of Victoria Institute on Aging and Lifelong Health, Stn. CSC Victoria, PO Box 1700, V8W 2Y2 Victoria, BC Canada; 4 School of Nursing, University of British Columbia, BC Cancer Suite 500, 686 West Broadway, V5Z 1G1 Vancouver, BC Canada

**Keywords:** Cancer, Cancer treatment access, Cancer treatment adherence, Health equity, Health disparities, Social determinants of health, Structural vulnerability, Vulnerable populations

## Abstract

**Background:**

A key pillar of Canada’s healthcare system is universal access, yet significant barriers to cancer services remain for people impacted by structural vulnerability (e.g., poverty, homelessness, racism). For this reason, cancer is diagnosed at a later stage, resulting in worse patient outcomes, a reduced quality of life, and at a higher cost to the healthcare system. Those who face significant barriers to access are under-represented in cancer control services Consequently, these inequities result in people dying from cancers that are highly treatable and preventable, however; little is known about their treatment and care course. The aim of this study was to explore barriers to accessing cancer treatment among people experiencing structural vulnerability within a Canadian context.

**Methods:**

We conducted a secondary analysis of ethnographic data informed by critical theoretical perspectives of equity and social justice. The original research draws from 30 months of repeated interviews (n = 147) and 300 h of observational fieldwork with people experiencing health and social inequities at the end-of-life, their support persons, and service providers.

**Results:**

Our analysis identified four themes presenting as ‘modifiable’ barriers to inequitable access to cancer treatment: (1) housing as a key determinant for cancer treatment (2) impact of lower health literacy (3) addressing social care needs is a pre-requisite for treatment (4) intersecting and compounding barriers reinforce exclusion from cancer care. These inter-related themes point to how people impacted by health and social inequities are at times ‘dropped’ out of the cancer system and therefore unable to access cancer treatment.

**Conclusion:**

Findings make visible the contextual and structural factors contributing to inequitable access to cancer treatment within a publically funded healthcare system. Identifying people who experience structural vulnerability, and approaches to delivering cancer services that are explicitly equity-oriented are urgently needed.

## Background

Widely acknowledged as a fundamental human right, access to healthcare is also a well-known determinant of health, and a key strategy to reduce health inequities [1, 2]. Despite the promise of “universal” healthcare access in Canada, significant inequities in access to healthcare persist. More specifically, inequitable access to cancer care in Canada is increasingly documented among structurally vulnerable population groups, including those experiencing intersecting impacts of poverty, unstable housing, and discrimination along the lines of race and gender [3–10]. In this paper, we employ the concept of ‘structural vulnerability’ which informs our understanding of processes and impacts of health and social inequities whereby populations or groups experience heightened vulnerability to poor health, because of their position in hierarchical relations of power, and the mutually reinforcing negative impacts of stigma, racism, poverty, and lack of access to social determinants of health [11–13].

Inequitable access to care is especially problematic in the cancer care sector, as early diagnosis and timely access to cancer treatment are linked with better health outcomes [7, 14]. Research indicates that cancer incidence is as much as four times higher among structurally vulnerable populations, who are also more likely to be diagnosed with advanced cancers and have worse cancer-related outcomes than more advantaged members of society [15–19]. Conversely, structurally vulnerable populations face significant barriers to accessing cancer care relative to their care needs. For example, there is less use of cancer control screening, prevention strategies, or primary care services for Canadians living in poverty or experiencing homelessness as a result of a constellation of barriers [6, 7, 17, 20–23]. Consequently, people are dying from cancers that are treatable or preventable, at a reduced quality of life [15–17, 19, 23].

Among structurally vulnerable populations, poor access to cancer treatment has been described in several countries that have publicly funded healthcare systems [4, 23–28]. This body of research indicates that groups experiencing socioeconomic disadvantage are: (1) more likely to experience delays in starting treatment (e.g., surgery, systemic therapy, radiotherapy); [23, 25, 26] (2) less likely to receive any treatment; [4, 5, 7, 23–25, 27−30] (3) experience poor adherence to radiation or systemic therapy(e.g., chemotherapy, hormone therapy, biologic therapy, immunotherapy); [7, 23] (4) receive care outside a dedicated cancer treatment facility; [23, 29] and (5) receive care that does not align with best practice guidelines compared to more advantaged members of society [23, 29]. While this research has largely focused on documenting cancer treatment disparities along the lines of sociodemographic differences, there is a need to better understand why these disparities exist and how social and structural determinants affect access to cancer care [9, 31−34]. Although some research has explored these issues, the focus has largely been on barriers to cancer *screening* [6, 7, 9, 21, 23, 35, 36], and *end-of-life care* [9, 23, 37] for structurally vulnerable populations, while little is known about barriers specific to cancer *treatment.* [6, 7, 23]

The cancer treatment trajectory is remarkably complex, and because of a growing emphasis on cost efficiency, treatment is primarily delivered through an outpatient model of care [38, 39]. This model of care requires patients to interface with diverse healthcare providers (e.g., oncology care providers, surgical specialists, and primary care providers), across multiple locations and healthcare environments (e.g., acute care services, cancer centers, and primary health clinics) [7, 38, 39], earning the description of a “broken”, “fractured” or “siloed” system [12, 33]. Cancer treatment itself is often multi-modal, and may involve a combination of surgical treatment, systemic therapy, and/or radiation therapy. To overcome the challenges of a fragmented cancer care system, a patient-centered approach to care, which places patient’s unique needs, preferences, and values at the center of a comprehensive and coordinated treatment plan, has increasingly been cited as a mechanism for improving the quality and continuity of care across the cancer care trajectory [4, 40−42]. However, these aims are based on the underlying assumption that patients have access to basic needs for daily survival; are linked to a social support network; and have the physical, psychological, and socioeconomic capacity to be able to self-manage their care needs and navigate a complex care system [1, 39, 42].

A predominant focus of biomedical treatment of cancer means that broader structural and social determinants of health that act as barriers to care remain unaddressed [10, 33]. Although Canada has been a leader in developing a theoretical foundation related to social determinants of health, it has been less successful in putting these concepts into action [43]–[44]. A key consideration is how unmet social determinants contribute to producing inequitable access to cancer care, and in turn, how these barriers produce differences in cancer outcomes.

### Aims

Theoretically informed by health equity (equal access for equal need) and critical social justice perspectives, this study aims to better understand barriers to initiating cancer treatment (e.g., surgery, systemic therapy, and radiation) and adherence to treatment (continuing with treatment as prescribed) for structurally vulnerable populations within the context of the Canadian cancer care system [2, 43, 45].

### Theoretical perspectives

Critical social justice and intersectional perspectives draw attention to how inequities in cancer outcomes are shaped by the social determinants of health (SDOH) and arise from systematic differences in access to essential resources [46]. The terms ‘inequities’ and ‘disparities’ in health are often used interchangeably to describe unfair or avoidable and possibly remediable differences in health, which are unjust and therefore morally concerning [45–47]. In efforts to redress health inequities, social and structural determinants of health perspectives reframe differences in health by focusing on the social, economic, environmental, political, and historical factors that produce conditions in which people are born, grow, live, work, and age [43, 48, 49]. These perspectives showcase how deficits in social and structural determinants of health rather than individual factors (e.g., behaviour, lifestyle) contribute to cancer- related inequities.

Grounded in Black feminist legal scholarship, intersectionality perspectives can be used to understand how aspects of a person’s social location create different modes of privilege or disadvantage in society [50, 51]. Intersectionality moves away from siloed and single categorization of socio-demographic groups (e.g., gender, race, class) to engage with multiple and intersecting power relations that produce complex, interdependent social inequities and forms of oppression [50, 51]. Poverty is particularly salient as it compounds vulnerabilities in health by structuring differential control over access to basic material resources, services, and opportunities [43, 48]. In this study, an intersectional lens has guided our exploration of how poverty and/or homelessness intersect with racism, sexism, experiences of trauma and violence, stigma in association with mental health issues or cognitive impairments, criminalization and substance related harms, and ableism produce barriers to care within the cancer care sector [43, 48, 52].

## Methods

We conducted a secondary analysis of data originating from a larger qualitative ethnographic study of the experiences of healthcare at the end-of-life for structurally vulnerable populations, originally published by Stajduhar and colleagues [12]. Data in the original study included: 300 h of participant observational fieldwork and 147 interviews with 25 people experiencing life-limiting conditions (e.g., advanced cancer, organ failure, severe COPD) alongside homelessness, poverty, racialization, and stigma; 25 supporters (e.g., street family, bio-legal family); and 69 service providers (e.g., housing and outreach workers, medical professionals). This secondary analysis is based on data from 16 participants experiencing structural vulnerability who were diagnosed with cancer. In addition, data excerpts from interviews and observations with supporters and service providers that were coded with the term ‘cancer’ have been included in our analysis. The participant demographics shown on Table 1 summarizes characteristics of participants experiencing structurally vulnerability as collected from the demographic questionnaire in the original study. Table 2 represents the cancer characteristics for structurally vulnerable participants, collated from the data excerpts and field notes.

**Table 1:** Structurally vulnerable participants’ characteristics (*n* = 16)

Characteristic		# of participants
Sex	Male	10
	Female	6
Age^a^	Average age	58
	Age range	50–81
Race/ethnicity^a^	White/European settler	9
	Indigenous identity	5
Sexual orientation^b^	Heterosexual	12
	LGBTQIA/2S	2
Marital status^b^	Divorced or separated	8
	Single	4
	Married or living in common-law	2
Source of income^b^	Disability	7
	Pension	5
	Employment insurance	1
	Social assistance	1
Housing status^a^	Social or public housing	6
	Market housing (with roommates/ financial supplements)	6
	Homeless (e.g., shelter, boat, hospital)	3
	Transitional (incl. hotel/motel)	1
Level of education^c^	Elementary school or less	2
	Middle school (grade 8)	1
	Some high school	1
	High school	5
	Some college (including trade school)	2
	College diploma	1
	University	1
Mental illness	Yes	9
	No	7

^a^Based on 15 participants. One participant did not answer this question.

^b^Based on 14 participants. One structurally vulnerable participant engaged in observation but died before completing a demographic form. One participants did not answer this question.

^c^Based on 13 participants. One structurally vulnerable participant engaged in observation but died before completing a demographic form. Two participants did not answer this question.

**Table 2:** Structurally vulnerable participants’ cancer characteristics (*n* = 16)

Characteristic		# of participants
Cancer site	Liver	4
	Lung	1
	Dual diagnosis: Lung and liver	1
	Head and neck	2
	Prostate	2
	Colorectal	1
	Anal	1
	Bone	1
	Adrenal	1
	Cervical	1
	Bone	1
	Skin	1
Stage at diagnosis^a^	III or IV	13
Location of diagnosis^b^	Emergency room or hospital	9
	Specialist office	1
Eligible for treatment at diagnosis?^c^	Yes	7
	No	3

^a^Based on 13 participants. The stage at diagnosis could not be determined from the primary data collected for three structurally vulnerable participants.

^b^Based on 10 participants. It could not be determined from the primary data the location of diagnosis for six of the participants.

^c^Based on 10 participants. It could not be determined from the primary data whether six participants were eligible for treatment at diagnosis.

### Data analysis

An interpretive thematic analysis was conducted, which aimed to describe barriers to cancer treatment for structurally vulnerable populations through the lenses of social justice perspectives and intersectionality theory. In our secondary analysis, the interview transcripts and field notes were compiled for each participant, along with relevant interview recounts from service providers and healthcare providers. Service providers’ notes were read separately and then together within the context of each participant. Each set of interview transcripts was analyzed in isolation and then together within the context of the ethnographic field notes. We began with an iterative process of re-coding data, and developing categories by identifying patterns in the re-coded data and expanding and then collapsing categories. In the final stages of analysis, we moved toward a more abstract, conceptual analysis to identify modifiable factors through the lens of health equity discourse and SDOH.

## Results

Our secondary analysis included sixteen participants with a cancer diagnosis (Table 1). Nearly all participants presented with advanced cancer at the time of diagnosis (n = 13) (Table 2). In addition, many had received their original cancer diagnosis in an emergency department or hospital (n = 9). Advanced stage diagnosis and comorbidities rendered some participants (n = 3) ineligible for treatment. This finding signals a need for further investigation of the social contexts surrounding barriers to early diagnosis. Among those with a clearly identified treatment plan, and for which treatment remained an option (n = 7), none of the participants completed their recommended course of treatment because of complex and intersecting social and structural factors, as described in our findings (below). Accordingly, our analysis indicates ‘missed opportunities’ to addressing the often less visible barriers to cancer treatment. We identified four themes in our analysis that highlight ‘modifiable’ (potentially remediable) barriers to accessing cancer treatment: (1) housing as a key determinant for cancer treatment; (2) impact of lower health literacy (3) addressing social care needs is a prerequisite for cancer treatment; and (4) intersecting and compounding barriers reinforce exclusion from cancer treatment.

### Housing as a key determinant for cancer treatment

Among the many challenges experienced by people living in poverty in our study, poor access to safe, secure, and stable housing presented as a significant barrier to cancer treatment. One quarter of the participants were found to be unstably housed and were living homeless in a transitional housing facility or emergency shelter. For participants who were homeless at diagnosis (n = 3), finding access to safe and stable housing was cited as their first priority before concerns for their cancer-related health could be addressed. Efforts to find housing caused delays in starting treatment, if cancer treatment was presented as an option. In some cases, unsuitable housing conditions, such as transitional housing or homeless shelters, meant that treatment was not offered by cancer specialists, because environmental conditions (e.g., crowded housing facilities, poor sanitation, shared bathrooms, lack of storage for medication) were deemed incompatible with safely managing potential side effects from treatment (e.g., risk of severe infection, nausea, diarrhea). According to healthcare workers and service providers, unsafe housing conditions meant that some participants could not be supported ‘in place’ to receive cancer treatment, as their increasing medical needs did not align with housing policies. To illustrate, a community health provider described the story of Ken^1^ who was living with advanced lung cancer on the third floor of a shelter with a broken elevator. He was unable to leave his building when shortness of breath prevented him from being able to walk up or down the stairs. Due to this precarious housing environment, he was considered ineligible for homecare or transportation services and could not physically access outpatient cancer treatment or supportive care. Service providers in our study regularly described cases where individuals living with cancer, and in need of medical treatment and support, were deemed too medically complex and thus ineligible for shelters or transitional housing, and yet seemed to have social needs too medically complex for the cancer treatment system. This example illustrates how neither system was able to accommodate both health *and* social care needs.

Barriers to cancer treatment also existed for those who had access to housing with medical support. However, in some cases policies led some participants to forgo their medically supportive housing, that enabled access to cancer treatment. For example, policies such as ‘single occupancy’ or ‘no visitor’ rules inadvertently removed participants away from their primary social networks and communities. This meant that their main caregivers (e.g., family or friends) were often unable to continue to provide in-home support. To illustrate, Betty was living outside her home community and estranged from her family. When she was diagnosed with advanced anal cancer, she moved from a single room occupancy hotel to a supportive housing facility where she was connected with home care services, and a meal program. Betty’s boyfriend was her main source of support; however, he rarely spent time with her at the supportive housing facility because of his own mobility issues and the facility’s no smoking and abstinence-based policies. As a result, Betty would visit him daily via public transit, where she experienced multiple falls that left her with broken ribs and a fractured hip. Despite the risks to her own health, she expressed the significance of these visits as they distracted her from her cancer symptoms. As she lost her physical independence due to progressive symptoms from her cancer, she began to experience severe loneliness despite living within a housing facility that supported her medical care needs.

Multiple evictions were also experienced by two participants during the course of their treatment journey due to a constellation of social and structural factors, including transitional housing policies, extreme poverty, substance use challenges and mental health challenges. Subsequently, these individuals were unable to continue their full course of recommended cancer treatment, as a result of unstable housing. To illustrate, Felix, as described in the observational field notes, was “*the classic case of what it means to fall through the cracks.*” Felix was diagnosed with cancer during an admission to hospital following a suicide attempt. He struggled with mental health and substance use challenges, and was otherwise homeless. Despite these challenges, Felix remained motivated to receive curative treatment for his cancer. The hospital staff advocated for Felix to stay longer than the regular length of stay, which enabled Felix to successfully complete an alcohol detoxification program, a requirement by his oncology care providers to start cancer treatment. Unfortunately, Felix experienced more challenges upon being discharged from hospital. The environment of the transitional housing facility he found placement was home to others who struggled with substance use. Under these circumstances, Felix began to experience his own difficulties with substance use, and he eventually became homeless again. Felix made every attempt to attend all his cancer related appointments; however, without a fixed address, telephone, or computer, it became increasingly more difficult for the cancer treatment facility to reach Felix regarding his appointments, and Felix became ‘lost to contact’. Felix’s case exemplifies how the structural conditions of poverty and unstable housing present as significant barriers to accessing health services. Notably, during his hospital stay, Felix could attend to both his cancer and mental health concerns when his social care needs were being met. Conversely, he was unable to complete his recommended course of treatment when the environmental conditions associated with precarious housing presented with barriers to achieving his optimal health status.

Finally, some participants described how they felt discriminated against for living in poverty and/or experiencing homelessness. For example, some participants were labelled as ‘non-compliant’ when they missed treatment appointments as a result of limited or no access to a phone, computer, or fixed address to receive mail. This also meant that participants had greater difficulty receiving notification of appointments or test results. In some cases, participants were discharged from treatment without being notified. These barriers and their impacts seem obvious (lack of access to a phone, for example). However, in the absence of an equity lens, and without an explicit orientation to the social determinants of health, the patients themselves seemed to be constructed as the ‘problem’, rather than the cancer care system. This highlights mismatches between how services are currently designed and delivered, in relation to the actual needs of people who are structurally vulnerable.

### Impact of lower health literacy

Cancer care is multifaceted and relies on patients’ ability to access and utilize the health care system. Our findings suggest lower health literacy is an overlooked barrier to cancer treatment. Within the context of our study, nearly all participants had identifiable lower levels of health literacy; yet, lower health literacy seemed to go unnoticed by oncology care providers. Consequently, participants were less enabled to make decisions, often at critical junctures throughout their cancer treatment journey. In our analysis, participants with lower levels of health literacy had less knowledge about their cancer and experienced greater difficulty processing written and verbal communication about critical information related to their cancer treatment plan. This translated to greater difficulty understanding: (1) their cancer treatment options; (2) appointment instructions or the need for particular tests (e.g., blood work, diagnostic imaging); (3) the purpose of recommended treatments; (4) how to self-manage treatment side effects; (5) how to navigate the cancer care system; and (6) were often reluctant to ask questions about their cancer care and treatment plan. These factors led to poor adherence to treatment (e.g., ‘skipped’ or ‘missed’ treatment), delays in receiving treatment, or caused some participants to forego treatment altogether.

During the treatment consultation phase, participants were less able to meaningfully engage in decisions related to their care plan when lower health literacy went unnoticed by their cancer care providers. Within the context of this study, some cancer care providers who used complex medical terms may not have been aware of their patients’ lower health literacy. This resulted in missed opportunities for patients to make informed decisions about their care and missed opportunities by healthcare providers to build health literacy skills by using accessible language. Subsequently, some participants did not receive treatment or complete their recommended course of treatment.

For example, mismatches in the delivery of health information led one participant to miss the critical window of opportunity for which treatment would be effective. Noah missed his window to receive life-prolonging chemotherapy for advanced rectal cancer. After multiple surgeries and radiation, Noah’s health took a drastic turn. He was meant to see an oncologist in the weeks following his second surgery for consideration of chemotherapy, however, this consult was delayed when he was hospitalized for anemia, rapid weight loss, and debilitating weakness. He initially understood his treatment options, yet he did not understand the time- sensitive nature of deciding on whether to pursue additional cancer treatment with chemotherapy:

“*And I didn’t feel like I got very well informed by the doctor there about my options. There was sort of a window of opportunity for taking the chemo. And really my slipping into kind of malnourishment was right in that window. And by the time I had gained that ten pounds and was thinking, okay maybe I can handle it now, that window was closed”.*

We identified that ‘missed’ opportunities to meaningfully engage with participants with lower health literacy (e.g., longer appointment times to build health literacy, using accessible medical language) also contributed to participants withdrawing during their cancer treatment consultations as complex medical concepts may have not been explained in a way that the participants could engage with. As Dani described his initial oncology consult: *“She explained to me she doesn’t know if the cancer started in the lungs and went to the liver, or the opposite way around… To me it’s confusing, because I don’t understand that. Like you’re telling me it just jumped over? How does this happen? Why only that lung?… There’s no sense her explaining it to me, because it will all be in medical jargon and I’ll never understand it. Plus, I’ll forget it five minutes after she tells me anyway*”. In this example, a mismatch in communication eventually led to mistrust in cancer care providers, and a reluctance to access cancer care.

During the treatment phase, participants with lower health literacy struggled with many components of an outpatient care model. To illustrate, outpatient cancer treatment requires patients to navigate across many different health environments and relies heavily on both verbal and written instructions. When appointment details for diagnostic imaging (e.g., CT scan, x-ray, MRI) and lab tests were not clearly understood, some participants did not complete important testing required for treatment, causing treatment delays. For others, inaccessible medical language meant that they did not understand the purpose or need for cancer treatment, which led to some participants foregoing treatment or discontinuing treatment early. For instance, Linda was being treated for advanced liver cancer while struggling with severe mental illness and substance use challenges alongside several other comorbidities. Despite these challenges, she remained on cancer treatment for several months. According to her healthcare provider, she did not have a clear understanding of the purpose of treatment, and one day and without a clear explanation from the participant, she decided to stop treatment for her cancer.

The expectations of an outpatient care model rely on a high degree of individual responsibility for self-management including: understanding and dealing with side effects, monitoring for more severe side effects, and knowing when to escalate their care to a cancer care provider. We identified that participants with lower health literacy experienced greater difficulty managing their care needs. For instance, Marleen struggled with severe nausea as a side effect from her chemotherapy, which led to rapid weight loss. She stated these side effects “knocked her out for weeks” after each treatment. Given she did not have a source of income; Marleen could not afford out-of-pocket expenses for her support medications or recommended food supplements. She and her partner also struggled with medical jargon and did not have a clear understanding of the purpose of cancer treatment, and stated she did “not like putting drugs into [her] body that could cause cancer”. Eventually, she discontinued treatment early, and reported they wanted to try natural means for treating cancer before undergoing more chemotherapy.

### Addressing social care needs is a prerequisite for cancer treatment

Although participants were facing a very real threat to their health from a cancer diagnosis, the competing priorities of daily survival such as access to stable housing, income support, nutritious food, and transportation were considered to be the first priority for many of the participants. With many unmet social needs, participants were especially vulnerable to the compounding challenges of navigating both the cancer system and the social system, which caused treatment delays or made it more difficult for participants to stay on the recommended course of treatment. This was particularly challenging for participants with lower health literacy levels, educational attainment, cognitive capacity, and mental health challenges. However, social care needs went largely unacknowledged and/or unaddressed by cancer care providers. This was related to a perception that addressing social care needs (including completing paperwork for social support services) was beyond their scope of clinical practice or their responsibility, and was rather a function of community-based healthcare services.

The barriers to accessing cancer treatment that we identified in our analysis are further compounded by system complexity and dis-integration. Participants especially struggled with obtaining access to social supports if they were not linked in with a primary care provider (e.g., family physician, nurse practitioner). In many cases, participants were left to navigate both the social care system and the cancer care system with minimal support. To illustrate, Marleen was initially diagnosed with advanced cervical cancer while staying as an inpatient in hospital. She was otherwise homeless and without an income. During her hospital stay, she and her partner struggled to obtain the social supports required for outpatient cancer treatment. She and her partner described how the hospital social workers seemed inexperienced at appropriately supporting people experiencing poverty, and other care providers did not attempt to help. To illustrate, a research assistant who interviewed Marleen shared: *They [Marleen and her partner] pulled out a stack of files with information they had been given about Marleen’s cancer, and about potential resources to access, and that they had been trying to navigate this information on their own, but a barrier was they didn’t have anyone to steer them in the right direction. They cited the issue with the bus pass as an example; they hadn’t fully understood whether they had to choose between the transportation subsidy and the bus pass, or whether they could get both, because the wording had been so ambiguous. And it would have been helpful to have someone figure it out and apply. They also said it would have been helpful to have someone to help them navigate all the issues around their tenancy dispute, and to help them find new housing. Preparing to move, they explained was their primary focus at the time.”*

In another example, George was diagnosed with advanced cancer and kidney failure as a result of his untreated cancer, after being taken to hospital against his will when his neighbors called a local police force. Over the course of his treatment George experienced challenges with unstable and unsuitable housing, poverty, and transportation that impacted his ability to attend appointments and receive care. According to field notes, George was unaware of the supports and services available to him. He did not recall if his cancer care providers offered information about assistance with transportation, a basic service offered by many cancer centers. When the broader context of patients’ lives and social care needs go unnoticed and/or action is not taken to address these basic needs, it renders them invisible.

### Intersecting and compounding barriers reinforce exclusion from cancer care

None of the participants completed their course of recommended treatment, primarily due to social disadvantage(s), unmet social determinants of health, and institutional policies that implicitly discriminated against individuals with greater social care needs. Importantly, these barriers were not experienced in isolation, but intersected with one another in ways that had the overall effect of excluding participants from cancer care. In our findings, we have used some examples from Marleen’s case to illustrate barriers to accessing cancer care; however, we revisit this case to exemplify how many of these barriers compound one another. With a diagnosis of advanced cervical cancer, Marleen experienced barriers to cancer treatment related to poverty and unstable housing. She was evicted from supportive housing during her chemotherapy treatments for advanced cervical cancer as the policies of the housing unit did not align with her increasing medical needs. Although she was on active cancer treatment, she was not well- supported in meeting her needs for housing and bounced from one temporary dwelling to the next, moving five times during treatment. As a result of unstable housing and the fact that she had no source of income, she and her partner did not have ready access to a phone, computer, or transportation, which made addressing their own unmet health and social care needs all the more challenging. When Marleen and her partner could not find housing in the city they were living, they chose to move to a remote location. As a result of poverty (including lack of transportation, phone or internet) and the isolated rural location, they encountered immense difficulty accessing care. Lower health literacy compounded these barriers, as Marleen and her partner were skeptical about the benefits of chemotherapy and did not seem to fully grasp the implications of delaying treatment, which were more challenging from a remote location. The intersections of these barriers to cancer treatment (lack of adequate housing, lack of appropriate transportation, lower health literacy, and ongoing unmet social needs) meant that Marleen was quite literally excluded from cancer care.

Moreover, past negative experiences in healthcare and experiences of stigma (often related to substance use and/or homelessness) and discrimination intersected with unmet social needs and a complex healthcare system, effectively pushing some participants outside the system. For example, Linda was living with advanced liver cancer as a result of untreated hepatitis C. She described repeated and ongoing negative healthcare experiences, substance use stigma, and a severe distrust for medical systems as one of the institutions that were supposed to help her, but did not. These barriers intersected with unstable housing, poverty, and lower health literacy. Linda received some chemotherapy after her cancer diagnosis but stopped treatment because she did not have a clear understanding of the purpose of treatment, felt her cancer should have been diagnosed earlier, and continued to experience discrimination and stigma.

## Discussion

Findings from our analysis suggest there are multiple and intersecting barriers in access to cancer treatment for structurally vulnerable populations. While we have intentionally focused on modifiable (thus potentially remediable) barriers to inform our discussion, our analysis reveals the compounding effects of structural inequities such as: stigma, discrimination, poverty, unstable housing, mental health and substance use challenges, insufficient access to transportation, food insecurity, and impact of lower health literacy. Additionally, inequities in access to cancer treatment are tremendously complex and often enmeshed, with barriers existing at the macro (social and structural determinants of health), meso (health system and cancer organizations), and micro (patient-provider and point-of-care interactions) levels that cannot be addressed by focusing on any one single determinant.

Our overarching analysis highlights how poverty and unmet needs for daily survival create significant barriers to accessing cancer treatment. At the macro level, poverty has been acknowledged as a root factor for other social determinants of health [8, 16, 35, 43, 48, 53–56]. Specifically, lack of access to safe and stable housing is a critical barrier to treatment eligibility or treatment completion. While addressing social needs such as housing and income is often viewed as outside the responsibility of cancer care organizations, it is an essential component to ensuring safe and equitable treatment delivery. These findings are consistent with recent research exploring the impact of housing among low-income cancer patients, which found that unclean or substandard living environments such as transitional housing or shelters were incompatible with managing the side effects of cancer treatment [55]. Within the context of siloed health and social care systems, there is an emerging call for policy- makers to employ ‘outside the box’ ways of tackling upstream determinants such as housing to improve health outcomes through inter-sectoral collaboration. An upstream approach to addressing material-based inequities and access to cancer treatment through direct action on the SDOH will require commitment and collaboration from cancer organizations and provincial and national governments, and community organizations. Healthcare providers within cancer care organizations also have a role to play in supporting individual patients, but also in advocating for health systems and structural change [33, 34].

Canada’s large geographic landscape presents further challenges, resulting in an inequitable distribution of healthcare resources. In this study, we found economic barriers (e.g., additional costs associated with travel, accommodation, and out- of- pocket expenses) related to treatment access were multiplied for those who live further away from a cancer treatment facility and led some participants to discontinue treatment when their social and healthcare needs were not consistently and purposefully addressed. Our findings are consistent with the literature, which suggests that geographical proximity is a key component of healthcare accessibility, and that healthcare resources must be easily reached and accommodated in a timely manner [1]. Based on an outpatient model of care, cancer treatment is typically delivered in dedicated cancer treatment facilities, primarily located in urban hubs. This model presents additional barriers for structurally vulnerable populations living in rural and remote communities, impacting treatment decisions and contributing to inequitable access to oncology consultations, systemic therapy, and radiation treatment [5, 7, 39, 57]. Notwithstanding, challenges with transportation can be reframed as a microcosm for much larger social issues associated with affordability and access to healthcare. Although transportation challenges are further deepened for those living in rural and remote communities, the geographies of ghettoization for structurally vulnerable populations living within urban environments have also been noted [48]. Albeit nuanced, our findings point to how an unreliable source of transportation increases vulnerability to accessing cancer treatment, regardless of urban and rural differences, and further highlight the cycles of oppression associated with living in poverty.

At the meso level, there is growing attention within healthcare organizations to improve the quality of healthcare delivery at the point of care [4, 40, 41, 58]. Similarly, patient-centered care has gained recognition in healthcare education, planning, and policy documents as a key pillar to providing high-quality healthcare [4, 40–42, 59]. However, our analysis suggests there is discordance between the rhetoric of patient-centered care and addressing the care needs of populations who experience significant social and health inequities. Our findings seem to suggest that significant social care needs (e.g., unmet social determinants, substance use challenges, lower levels of health literacy) may go *unidentified* and subsequently *unaddressed* within the context of cancer treatment. Within the context of a cancer treatment plan, social needs were not consistently prioritized, compared to biomedical needs. Notably, for those participants who were offered treatment (n = 7), none of their treatment plans included a systematic approach to addressing their social care needs, and consequently, none of the participants were able to complete the recommended course of treatment. Our findings also demonstrate how some participants discontinued treatment early or became ‘lost to follow- up’ when left to navigate a siloed cancer care and social care system without consistent support from cancer care providers. In an effort to improve equitable access to cancer services and to bridge gaps across the patient care trajectory, the concept of ‘patient navigators’ have been taken up by many cancer care organizations across Canada [60]. However, the role of patient navigators has not been systematically implemented across all settings, and factors into one piece of a complex puzzle [60]. Within the context of multiple or changing cancer care providers, we found that no singular healthcare professional was identified as the ‘most responsible provider’ for coordinating social supports such as access to suitable housing, nutritious food, transportation services to and from appointments, and coverage for out-of-pocket medical expenses necessary for treatment (e.g., anti-nausea medication). Although programs to address some of these needs do exist, support and coordination to access them is required. For example, the Manitoba Home Cancer Drug Program covers medications used to treat cancer or support the treatment of cancer, but requires patients to be covered under provincial health insurance, and then registered with Manitoba Health’s Pharmacare program [61]. This also requires patients to have a home address to complete the application forms. Although helpful, careful attention is needed to how these supports may still be inaccessible for people experiencing structural vulnerability.

Moreover, our findings revealed the timeliness of facilitating access to social care needs was of significant importance in enabling participants to adhere to their treatment plans. In other words, until participants’ social care needs were met, they could not reasonably attend to their cancer diagnosis or engage in cancer treatment. A fundamental shift is required in the design and delivery of cancer care that also prioritizes social care needs is required in order to facilitate equitable access to cancer treatment.

Paradoxical to patient-centeredness, there has been a shift towards an outpatient treatment model, alongside a shift in discourses of patient engagement from recognizing the importance of patient involvement in care to expectations that patients are responsible for ‘self -managing’ or directing their own care [59, 62]. Self-management discourses put responsibility on patients to manage their own care needs including (1) monitoring and managing treatment side effects (2) deciding how or when to escalate care needs (3) managing multiple medications and appointments (4) and psychological, social, and financial supports [41, 59]. Moreover, patients are increasingly positioned as the primary cancer treatment decision maker, rather than participating in a shared decision making model [62]. While a self-management model of care has facilitated managing high volumes of patients with cancer, it is somewhat problematically geared towards a highly supported, self-directed, and educated patient demographic [41, 59]. Yet, considering research suggests that the expectations of self-managed cancer care even challenge well-educated, high income, and supported patients [59, 62]. These challenges are all the more amplified for structurally vulnerable populations. In our study, we found that lower health literacy, mental health and substance use challenges impacted participants’ agency to meet the basic threshold for outpatient care. In some cases, this led to participants discontinuing treatment early. This is consistent with the findings of a scoping review documenting the impact of lower levels of health literacy in the context of cancer care among homeless adults, which found that those with lower health literacy have less knowledge about their cancer and treatment, significantly influences the ability to self-manage and access to cancer care [56]. Notably, our research demonstrates that cancer treatment models based on self-management may inadvertently exclude people experiencing structural vulnerability and/or those with significant social care needs such as lower levels of health literacy.

Finally, we found significant mismatches in the way cancer care services are designed and delivered and the actual needs of people who are structurally vulnerable which serve to reinforce and perpetuate existing health and social inequities. To meaningfully address the barriers to accessing cancer treatment that we identified, a major shift in the way cancer care services are designed and delivered at the point of care is required. As one potential avenue forward, equity-oriented healthcare is an approach to improving care that focuses on reducing the impacts of structural inequities (e.g., poverty, stigma, unstable housing), and the persistent mismatches between current approaches to healthcare and the actual needs of people experiencing structural vulnerability by informing organizational-level changes in how services are designed and delivered [63]. Browne and colleagues’ approach to transformative equity-oriented healthcare is founded on three key dimensions: **(1)** trauma- and violence-informed care, **(2)** culturally safe/anti-racist care, and **(3)** harm reduction philosophies with specific attention to mitigating the harms of substance use stigma [63]. Importantly, these dimensions have an explicit focus on building trust, fostering safety, and creating a welcoming environment for people experiencing structural vulnerability.

Through the integration of equity-oriented healthcare approaches, healthcare organizations can take direct action on social determinants of health and improve access to care through policy changes that accommodate the needs of those who may not fit within the normative patient population [33, 63]. For example, taking an equity-oriented approach to cancer treatment services would mean that social care needs are identified early and addressed through interdisciplinary team-based and wrap-around care so that cancer treatment is accessible and feasible [22, 33, 34]. Moreover, policies would be revisited to identify how care pathways could become more tailored, flexible, and accommodating to individual patient contexts. For example, organizational or unwritten policies that limit appointment lengths or result in ‘consequences’ for missed appointments should be examined and revised to take into account the intersecting barriers to access already experienced by structurally vulnerable populations. Finally, with its emphasis on fostering trust and safety, equity-oriented healthcare approaches would be one pathway to addressing an important finding from this secondary analysis—that establishing trusting patient-provider relationships was of utmost importance for those who had experienced any kind of abuse, past or present trauma, or discrimination.

### Limitations

The findings from this secondary analysis should be considered within the context of several limitations. First, the primary study, from which we drew our data, was focused on barriers to palliative care for structurally vulnerable populations, rather than specifically focused on barriers to cancer treatment. However, within the primary data, over half (60%) of the sample population had a diagnosis of advanced cancer and provided a robust source of data on barriers to cancer treatment. Secondly, some of the data raised questions about potential barriers to accessing cancer treatment, which we were not able to fully explore within the context of a secondary analysis. Future primary research is needed to more fulsomely explore specific barriers to cancer treatment for this population.

## Conclusion

This secondary analysis explored barriers to cancer treatment for structurally vulnerable populations within the context of the Canadian cancer care sector, highlighting how the many compounding unmet social determinants and structural forces limit opportunities and create barriers in accessing cancer treatment within a universal healthcare system. Although we have explored barriers specific to Canada, our findings can be applied to other countries with publicly funded access to cancer care, and for which inequities in accessing cancer treatment have been widely acknowledged. Importantly, we identified that addressing less visible social care needs for populations experiencing structural vulnerability was an essential pre-requisite in one’s ability to attend to their cancer diagnosis. In particular, the effects of poverty and a lack of access to safe and stable housing have been identified as salient upstream determinant. An examination of barriers to cancer treatment with greater attention to housing legislation, and specific health care policies and practice could inform the next steps towards redressing inequities in access to cancer treatment and outcomes. Finally, integrating equity-oriented principles into new models of care informed by people with lived experience is a potential way forward.

## Data Availability

Because of privacy and ethical considerations, the data are not publicly available. Inquiries regarding the original dataset can be directed to KS.

## Footnotes

1 To protect the identity of participants, pseudonyms have been used throughout.

## Abbreviations

SDOH: Social determinants of health
